# Chitosan-glucose derivative membrane obtained by Maillard reaction improves cartilage repair in a rabbit model

**DOI:** 10.1186/s13018-024-05127-7

**Published:** 2024-10-05

**Authors:** Po-Yao Chuang, Shun-Fu Chang, Ying-Chen Lu, Kuo-Chin Huang

**Affiliations:** 1 College of Medicine, Chang Gung University, Taoyuan, Taiwan; 2 Department of Orthopaedic Surgery, Chiayi Chang Gung Memorial Hospital, No. 6, West Sec., Chia-Pu Rd., Pu-Tz City, Chiayi County 61363 Taiwan; 3 Department of Medical Research and Development, Chiayi Chang Gung Memorial Hospital, Chiayi, Taiwan; 4 Department of Food Science, National Chiayi University, Chiayi, Taiwan

**Keywords:** Chitosan, Maillard reaction, Osteochondral defect, Cartilage, Biomaterial

## Abstract

**Background:**

Treatment of articular cartilage injury remains a challenging clinical problem in orthopedics. Chitosan-derived biomaterial could be a potential adjuvant treatment to improve cartilage repair. In the current study, we examined the effects of two potential chitosan-derived materials on cartilage regeneration of osteochondral defects in rabbits.

**Methods:**

An osteochondral defect was created over the rabbit knee and treated using three approaches: group A received no material (*n* = 24), group B received chitosan membranes with glucose absorption (CGA; *n* = 25), and group C received chitosan-glucose derivative membranes obtained via the Maillard reaction (CGMR; *n* = 25). Cartilage repair over the osteochondral defect was analyzed 12 weeks post-surgery via histological analysis, immunostaining, and reverse transcription-qualitative polymerase chain reaction (RT-qPCR) for type-I and type-II collagen mRNA.

**Results:**

According to histological analysis, CGMR-treated defects showed significantly improved modified O’Driscoll scoring when compared with no material- and CGA-treated defects (20.9 ± 4.3 vs. 13.00 ± 2.5 and 17.7 ± 4.6, *p* < 0.001). Moreover, group C exhibited higher intensity of type-II collagen immunohistochemical staining over the regenerated cartilage than groups A and B, along with increased expression of type-II collagen mRNA by RT-qPCR.

**Conclusions:**

CGMR might improve cartilage regeneration in osteochondral defects.

## Background

Chitosan is a deacetylated derivative of chitin, primarily extracted from the exoskeleton of crustaceans, insects, and fungi. Chitosan has received considerable attention owing to its unique bioactive properties, such as biodegradability, biocompatibility, hydrophilicity, antimicrobial properties, anti-inflammatory properties, and tissue healing effects [1]. Chitosan is a linear natural carbohydrate biopolymer with a structure that shares certain characteristics with glycosaminoglycan and hyaluronic acid, both present in articular cartilage [2]. It has a hydrophilic surface with a high hydrophilic capacity; therefore, it may attract and maintain fluids and cells and further promote cell adhesion and proliferation in tissue defects [3, 4]. Based on these properties, chitosan can be deemed a satisfactory candidate in the field of tissue engineering and bone and cartilage regeneration [5, 6]. However, as its designed degree of acetylation typically ranges between 5 and 40% for commercial products, chitosan is only soluble under acidic conditions, thereby limiting its application. Various studies have focused on modifying the aqueous solubility or improving the physicochemical properties of chitosan. For example, glycosylation, which is the introduction of oligosaccharide side chains into the chitosan backbone, could be a promising approach to overcome this limitation [7]. Glycosylated-chitosan derivatives exhibit substantially improved solubility at a neutral or basic pH, in addition to higher metal-ion chelating capacity, antibacterial activity, and antioxidant capacity than native chitosan [8–10]. Maillard reaction (MR) is a non-enzymatic chemical reaction that involves the condensation of a nitrogenous compound and a carbonyl group of a reducing sugar under heat treatment [11]. MR is a popular approach for synthesizing glycosylated-chitosan derivatives and has been largely characterized by Chung et al. and Gullón et al. [8, 12].

One known application of chitosan is the treatment of cartilage defects. Articular cartilage is a specialized tissue that covers joint surfaces, and its structure and function can be compromised by traumatic or degenerative joint diseases. However, treating cartilage defects can be challenging, given that damaged cartilage tissue has a limited capacity for self-healing owing to its avascular nature. Microfracture, also known as bone marrow stimulation, is a relatively simple and commonly used surgical treatment for localized cartilage defects. During this procedure, the subchondral bone below the cartilage lesion is perforated to allow bone marrow-derived stem cells to migrate to the defective site and induce a reparative response [13]. Different strategies, including the implantation of cellular or acellular biomaterials, have been applied to improve the outcome or quality of repaired cartilage after microfracture. Acellular biomaterials offer various advantages when compared with cellular biomaterials, such as the lack of donor-site morbidity, absence of cell culture costs, readymade availability, and simplified surgical procedures [14]. The implantation of acellular biomaterials for cartilage defects, including natural chitosan and its derivatives, has been extensively explored [15, 16].

Glycosylated-chitosan is a potential biomaterial capable of improving cartilage repair after microfracture induction for treating cartilage defects, owing to its superior physicochemical properties. Lactose-modified chitosan has been shown to induce cell aggregation upon contact with a primary culture of pig chondrocytes, stimulating the production of aggrecan and type-II collagen [17]. Key parameters that influence the MR, including the concentration and molecular weight of chitosan, glucose concentration, heating temperature, and reaction times, have been comprehensively clarified in subsequent studies [10, 12]. In contrast to MR, crosslinking between chitosan and glutaraldehyde yields copolymer materials with a porous network and improved adsorption properties [18]. A chitosan-glucose complex achieved by glucose absorption was found to enhance the proliferation of both human osteoarthritis and SW1353 chondrocytes by regulating the mammalian target of rapamycin complex 1 (mTORC1) signaling [19]. Herein, we examined the effectiveness of chitosan membranes with glucose absorption (CGA) and chitosan-glucose derivative membranes obtained by MR (CGMR) on osteochondral defects in a rabbit model. While the Microfracture technique is commonly used for full-thickness cartilage defects to stimulate bone marrow-derived stem cells, our study focuses on osteochondral defects to explore the potential for cartilage restoration using this model. To the best of our knowledge, this is the first in vivo study investigating these two chitosan derivative materials for treating cartilage defects.

## Methods

### Fabrication of CGA

Two grams of chitosan (low molecular weight; Sigma-Aldrich, St Louis, MO, USA) was dissolved in 1% (w/v) acetic acid under continuous magnetic stirring at 200 rpm and 65 °C for 2 h to obtain a final concentration of 2% (w/v) chitosan solution. After filtration, the chitosan solution was mixed in a 24:1 ratio with 0.125% (v/v) glutaraldehyde (Sigma-Aldrich) for the crosslinking reaction, and 5 mL of the solution was poured into a 6 cm dish and oven-dried at 55 °C for 12 h to obtain a chitosan membrane. The chitosan membranes were washed thrice with ethanol and six times with deionized water. Finally, the membranes were immersed in 25 nM glucose solution for 24 h to obtain CGA membranes and exposed to ultraviolet C light for 15 min before use.

### Fabrication of CGMR

The CGMR was synthesized according to the method described by Gullón et al. [12] with some modifications. Briefly, the pH of the 2% (w/v) chitosan solution was adjusted to 5.5 by the slow addition of 0.1 M aqueous NaOH. Then, glucose was added to the resulting solution while stirring to obtain a final concentration of 2% (w/v). MRs were performed at 60 °C by shaking at 100 rpm for 24 h; reactions were suspended by placing the test tubes in an ice bath for 10 min. Finally, 5 mL of the resulting solution was poured into a 6 cm dish and then oven-dried at 55 °C for 4 h to obtain the CGMR.

### Animals and experimental design

All animal experiments complied with the ARRIVE guidelines and were performed following the U.K. Animals (Scientific Procedures) Act, 1986. All animal experiments were approved by the Institutional Animal Ethics Committee of Chiayi Chang Gung Memorial Hospital (Approval No. 2018092603). Male New Zealand white rabbits (*n* = 37, 74 knees) between six and eight months old were used in the current study. Three materials (no material, CGA, CGMR) were implanted in the opposite knees of the same rabbit to mitigate the effects of animal variation. Three different groups were established, with the following number of knees covered by the material over the osteochondral defect per group: group A, no material, *n* = 24; group B, CGA, *n* = 25; and group C, CGMR, *n* = 25. Finally, 14 knees in each group were used for further histological and immunohistochemical analyses. Ten knees in group A and 11 knees in groups B and C, respectively, were used for RNA isolation and reverse transcription-qualitative polymerase chain reaction (RT-qPCR).

### Membrane implantation in osteochondral defects in rabbit knee joints

The rabbits were anesthetized by intramuscular injection of tiletamine/zolazepam (12.5 mg/kg; Zoletil 50™, Vibrac Laboratories, Carros, France) and xylazine (5 mg/kg). Maintenance was achieved via inhalation of isoflurane, nitrous oxide, and oxygen mixture. Prophylactic antibiotics (procaine penicillin, 10 mg/mL) and analgesics (ketoprofen, 2 mg/kg) were administered intramuscularly prior to surgery. Rabbit knees were prepared by shaving the hair and disinfecting the site with betadine and 70% ethanol scrubs. The surgical procedure was identical for all subjects and was performed by a single surgeon.

A midline skin incision and medial parapatellar incision were made to open the joint capsule and enter the knee joint. After the knee was flexed with patellar lateral retraction, the articular cartilage surface over the medial femoral condyle was exposed. A cartilage defect was created by drilling with a 2 mm drill to a depth of 2 mm, and subchondral bone exposure with bleeding was confirmed. In group A, the cartilage defects did not receive a material implant. In groups B and C, cartilage defects were covered with CGA and CGMR, respectively. The covering membranes were obtained using a 2 mm skin punch biopsy needle, were round in shape, and 2 mm in diameter. The membranes were rinsed with blood from the subchondral bone, placed, and impacted over the cartilage defects. Thereafter, the knees were cycled through a range of motions to ensure satisfactory fixation and membrane sealing. The patella was relocated, and the wounds were closed with a silk suture layer by layer, including the capsule and skin, in a simple interrupted suture pattern. After wound closure, we tested the motion range of the knees and confirmed that there was no joint instability or patellar dislocation. Thereafter, bacitracin-neomycin ointment was applied to the wound, and the rabbits were returned to their cages after regaining consciousness. Postoperative antibiotics were administered intramuscularly twice daily for one day post-surgery. All rabbits were allowed full weight-bearing and unconstrained movement postoperatively. Regular checks were performed to assess feed intake and limb movement. All animals were housed indoors in pens during the remaining study period.

### Retrieval of joints after 12 weeks of implantation

Animals were humanely sacrificed 12 weeks postoperatively using a lethal dose of Zoletil 50™ (50 mg/kg) and xylazine (23 mg/kg). The knee joints were opened and examined for signs of inflammation or infection. The distal femora, including the medial and lateral condyles, were resected *en bloc* and retrieved. The specimens were divided into three groups based on treatment and were directly processed for RNA isolation or stored in 10% formalin for paraffin embedding.

### Histological analysis

After fixation with 10% buffered formalin, the distal femora were decalcified with a 10% ethylenediaminetetraacetic acid (EDTA) solution and dehydrated in ethanol. The medial condyles were infiltrated and embedded in paraffin. Sections were prepared sagittally as 5 μm segments, stained with hematoxylin and eosin (H&E) and Safranin-O stain. The defect sites and regenerated cartilage were observed under a light microscope (Olympus, BX51, Melville, NY, USA) and scored using the modified O’Driscoll scoring method based on the following parameters: cellular morphology, structural characteristics, freedom from cellular degenerative changes over the defect site and adjacent cartilage, subchondral bone reconstitution, repair cartilage bonding to the subchondral bone, and Safranin-O staining presentation (Table 1). The histological scores are represented as the average ± standard deviation (SD) of replicates.

**Table 1:** Modified O’Driscoll Histology score

Characteristic	Grading	Score
I. Cellular morphology		
Hyaline cartilage %	80–100%	8
	60–80%	6
	40–60%	4
	20–40%	2
	0–20%	0
II. Structural characteristics		
A. Surface irregularity	Smooth and intact	2
	Fissures	1
	Severe disruption, fibrillation	0
B. Structural integrity	Normal	2
	Slight disruption, including cysts	1
	Severe lack of integration	0
C. Thickness	100% of normal cartilage	2
	50–100% or thicker than normal	1
	0–50%	0
D. Bonding to adjacent cartilage	Bonded at both ends of the graft	2
	Bonded at one end/partially at both ends	1
	Not bonded	0
III. Freedom from cellular changes of degeneration	Normal cellularity, no clusters	2
	Slight hypocellularity, < 25% chondrocyte clusters	1
	Moderate hypocellularity, > 25% clusters	0
IV. Freedom from degenerate changes in adjacent cartilage	Normal cellularity, no clusters, normal staining	3
	Normal cellularity, mild clusters, moderate staining	2
	Mild or moderate hypocellularity, slight staining	1
	Severe hypocellularity, slight staining	0
V. Reconstitution of subchondral bone	Complete reconstitution	2
	> 50% reconstitution	1
	≤ 50% reconstitution	0
VI. Bonding of repair cartilage to *de novo* subchondral bone	Complete and uninterrupted	2
	50–100%	1
	< 50%	0
VII. Safranin-O staining	> 80% homogeneous positive stain	2
	40–80% homogeneous positive stain	1
	< 40% homogeneous positive stain	0
Total score		27

### Immunostaining

The histological sections were deparaffinized with xylene (3803665; Leica Biosystems, Buffalo Grove, IL, USA) and rehydrated in alcohol (3803686; Leica Biosystems). Heat-induced epitope retrieval was performed by submerging slides into preheated antigen retrieval citrate solution (CBB500; ScyTek, Logan, UT, USA) and steaming for 20 min. Sections were incubated in Hydrogen Peroxide Block (TA-060-HP; Thermo Fisher Scientific, Waltham, MA, USA) for 10 min, then Protein Block (TA-060-PBQ; Thermo Fisher Scientific) for 10 min after twice washing with phosphate-buffered saline and 0.1% Tween^®^ 20 Detergent. Subsequently, the sections were incubated with primary antibody, anti-collagen II (#7005; Chondrex, Redmond, WA, USA) at 1:100 dilution for 40 min and secondary antibody, Peroxidase AffiniPure Goat Anti-Mouse IgG (H + L) (115-035-116; Jackson ImmunoResearch, Suffolk, UK) for 30 min. Sections were developed with 3,3′-Diaminobenzidine (DAB) (TA-060-QHSX; Thermo Fisher Scientific) for 3 min and finally counterstained with hematoxylin (3801522, Leica).

### RT-qPCR for type-I and type-II collagen mRNA

Briefly, the regenerated cartilage was excised, ground, and homogenized. The naive cartilage from the lateral femoral condyle of the same knee, distant from the osteochondral defect, was used as the control. Total RNA was isolated using TRIzol^®^ Reagent (15596026, Thermo Fisher Scientific) following the manufacturer’s instructions. Complementary DNA was synthesized using the RevertAid First Strand cDNA Synthesis Kit (Thermo Fisher Scientific), according to the manufacturer’s instructions. Quantitative PCR was performed using the CFX96 Touch Real-Time PCR Detection System (Bio-Rad, Hercules, CA, USA). A total volume of 25 µL reaction mix, containing 12.5 µL SYBR^®^ Green Master Mix (2X) (Bio-Rad), 1.0 µL primer pairs, 5 µL template, and 6.5 µL dH_2_O was used for the reaction. The amplification procedure was as follows: polymerase reaction and DNA denaturation at 95 °C for 5 min; amplification at 95 °C for 10 s and 58 °C for 30 s for 45 cycles. Beta-actin was used as the housekeeping gene to normalize the expression of the target gene, and relative gene expression was expressed as the fold change using the 2^−ΔΔCt^ method.

The oligonucleotide primers were based on sequences available from GenBank (National Center for Biotechnology Information, National Library of Medicine, National Institute of Health, Bethesda, Maryland). Primers for type-I collagen were constructed from the 5595 bp sequence in the rabbit collagen type-I alpha 1 chain (COL1A1) mRNA. The forward primers were constructed from bases 2032–2050 with the sequence 5’-GATGGTGCTAAGGGTGATG-3′, and reverse primers were constructed from bases 2164–2146 with the sequence 5’-GACCAGTTTCACCTCTGTC-3′. Primers for type-II collagen were based on 4788 bp sequences in collagen type-II alpha 1 chain (COL2A1) mRNA. The forward primers for type-II collagen were constructed from bases 523–541 with sequence 5’-CTTGGTGGAAACTTTGCTG-3′, and reverse primers were constructed from bases 601–583 with the sequence 5’-CTTGCATCACTCCATC-3′. Actin was used as an internal control, and the upstream primers were constructed from bases 1258–1276 with the sequence 5’-CAGAAACGAGACGAGATTG-3′, and downstream primers were constructed from bases 1355—1337 with the sequence 5’-AAATCCTGAGTCAAAAGCG-3′.

### Statistical analysis

Data were presented as mean ± SD. SPSS software (version 20.0 for Windows; SPSS Inc., Chicago, IL, USA) was applied for data management and statistical analysis. Statistical analysis was performed by using a 1-way analysis of variance (ANOVA) followed by Scheffe’s test. Any difference with *p* < 0.05 was considered statistically significant.

## Results

### Histological scoring of regenerated cartilage 12 weeks post-implantation

**Fig. 1:**
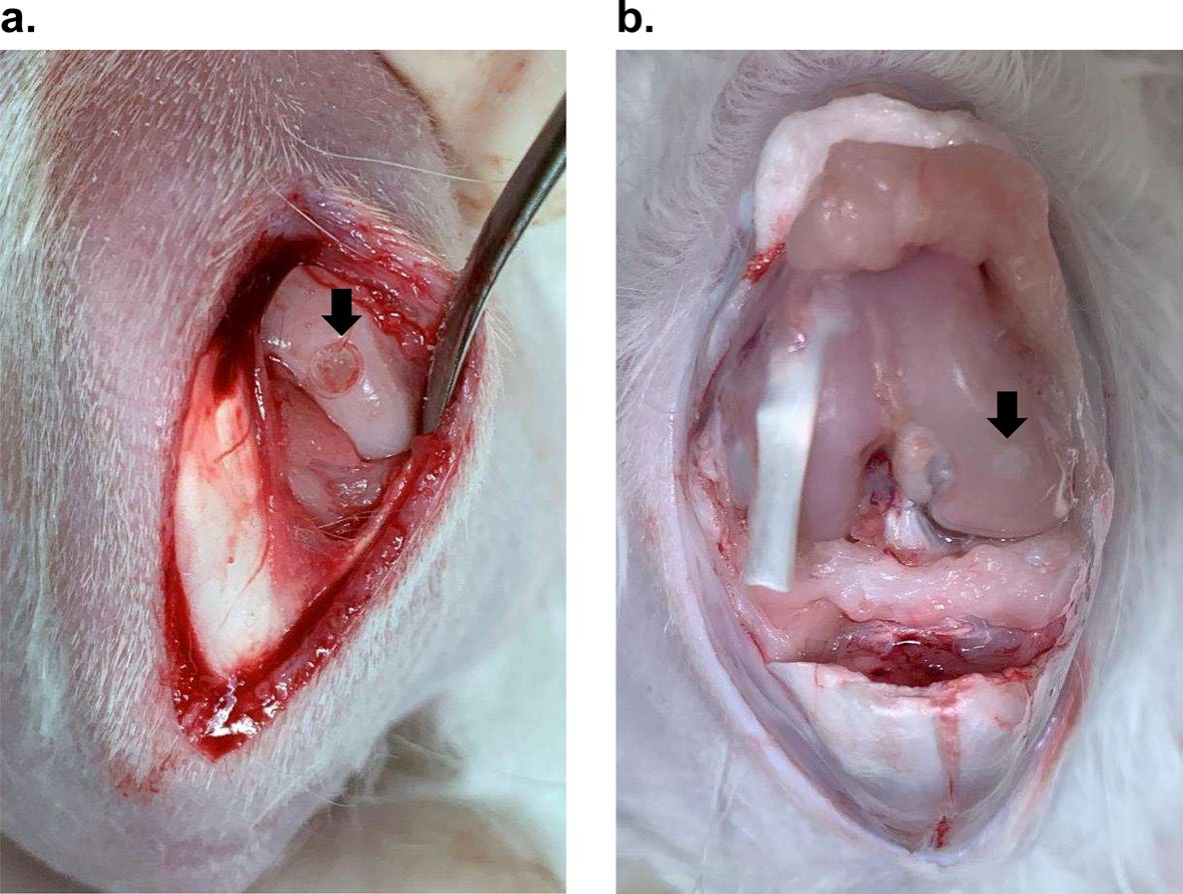
Gross photograph of the osteochondral defect surgically created in rabbit knees. (**a**) The knee joint was partially opened, and a 2 mm-diameter osteochondral defect was created on the medial femoral condyle, as indicated by the arrow. (**b**) Gross morphology of retrieved joints 12 weeks post-implantation. The arrow indicates the regenerated cartilage

**Fig. 2:**
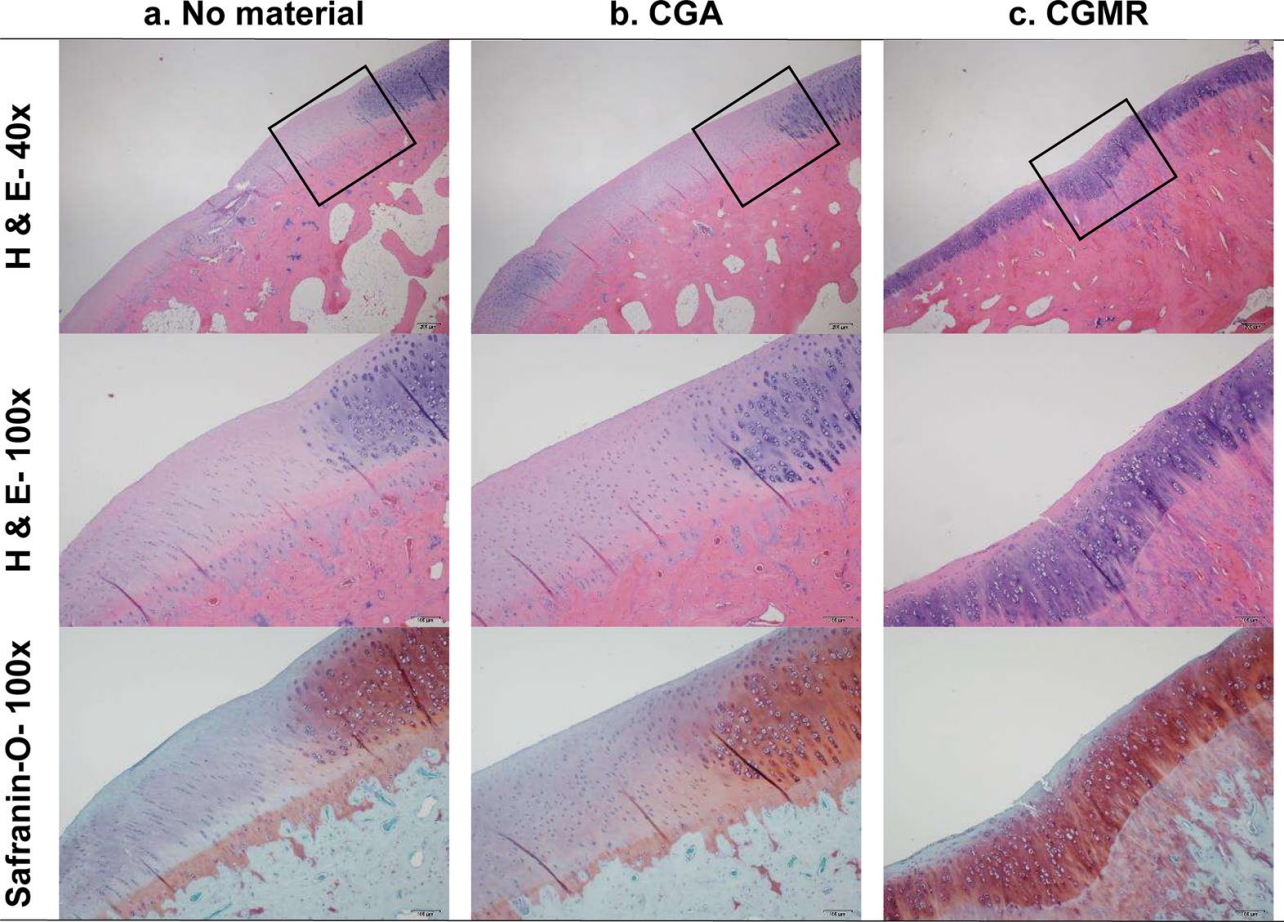
Histological images of regenerated cartilage subjected to hematoxylin and eosin and safranin-O staining. The regenerated cartilage in group C exhibits superior histological structure and cellular morphology, with more homogeneously positive safranin-O staining than that in the other groups

**Fig. 3:**
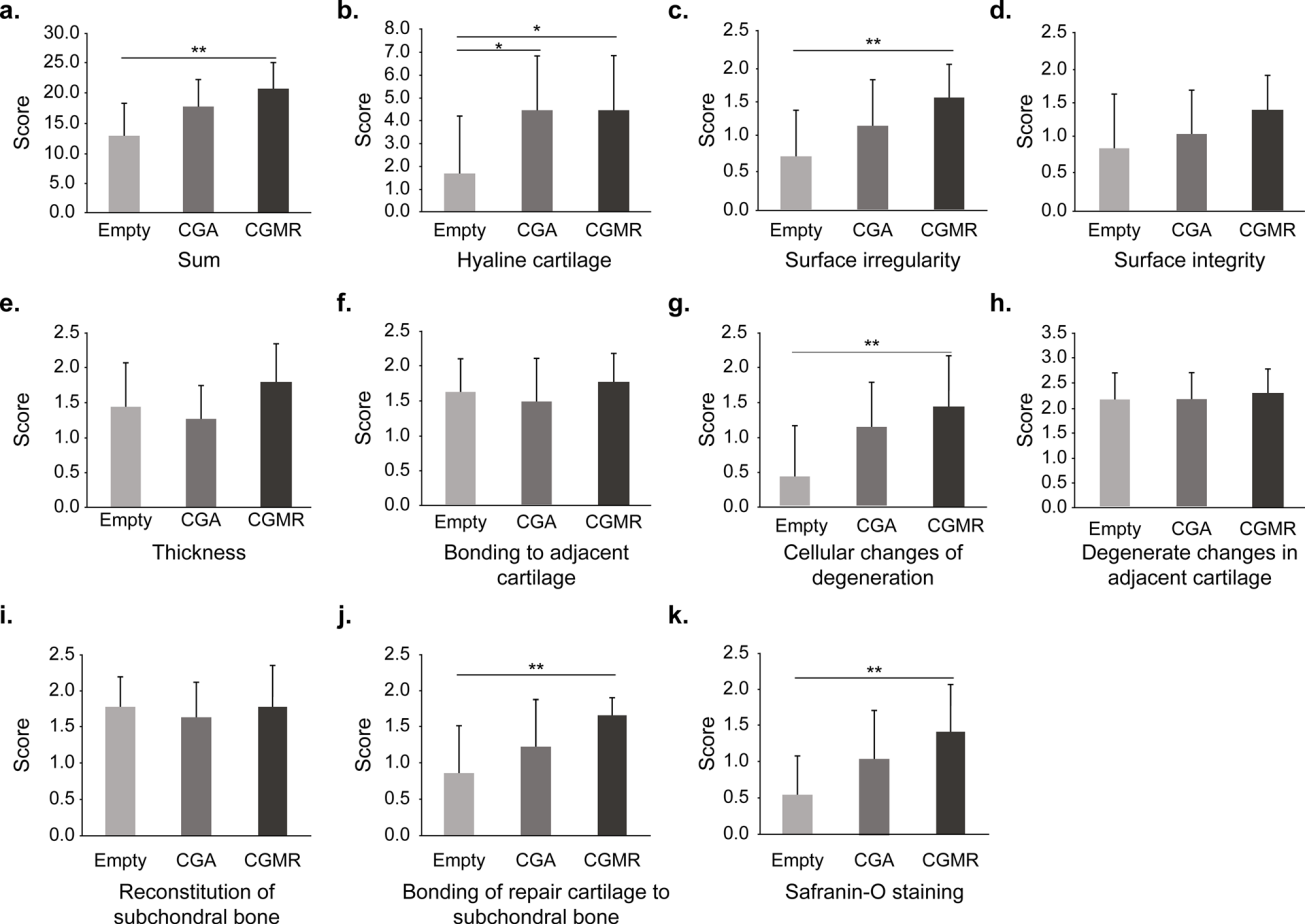
Modified O’Driscoll histology score of the regenerated cartilage. (**a**) A significant difference is observed between groups A and C. (**b**)–(**k**) Scores for various characteristic categories. The histological scores are presented as the average ± standard deviation (SD) of replicates. Significance is denoted by *p < 0.05 and **p < 0.01

Gross images of the osteochondral defect and cartilage regeneration are shown in Fig. 1. A modified O’Driscoll scoring system was used to assess the features and quality of the regenerated cartilage tissue at the defect site [20]. Representative histological images of H&E- and Safranin-O-stained sections are shown in Fig. 2. Groups B and C showed a trend toward an increased O’Driscoll score. The total histological scores of groups A, B, and C were 13.00 ± 2.5, 17.7 ± 4.6, and 20.9 ± 4.3, respectively, with a significant difference in scores observed between groups A and C (*p* < 0.001); however, differences between groups A and B or B and C were not statistically significant. Group C had higher scores in terms of hyaline cartilage, surface irregularity, freedom from cellular changes of degeneration, bonding of repair cartilage to *de novo* subchondral bone, and Safranin-O staining than groups A and B (Fig. 3). Accordingly, CGMR could improve the quality of regenerated cartilage by inducing a high proportion of hyaline cartilage and improving surface structure without impacting the surrounding environment, including adjacent original cartilage or subchondral bone. Degeneration over the adjacent cartilage was not noted owing to the relatively short postoperative period (12 weeks).

### Immunohistochemistry and RT-qPCR of type-I and type-II collagen mRNA

**Fig. 4:**
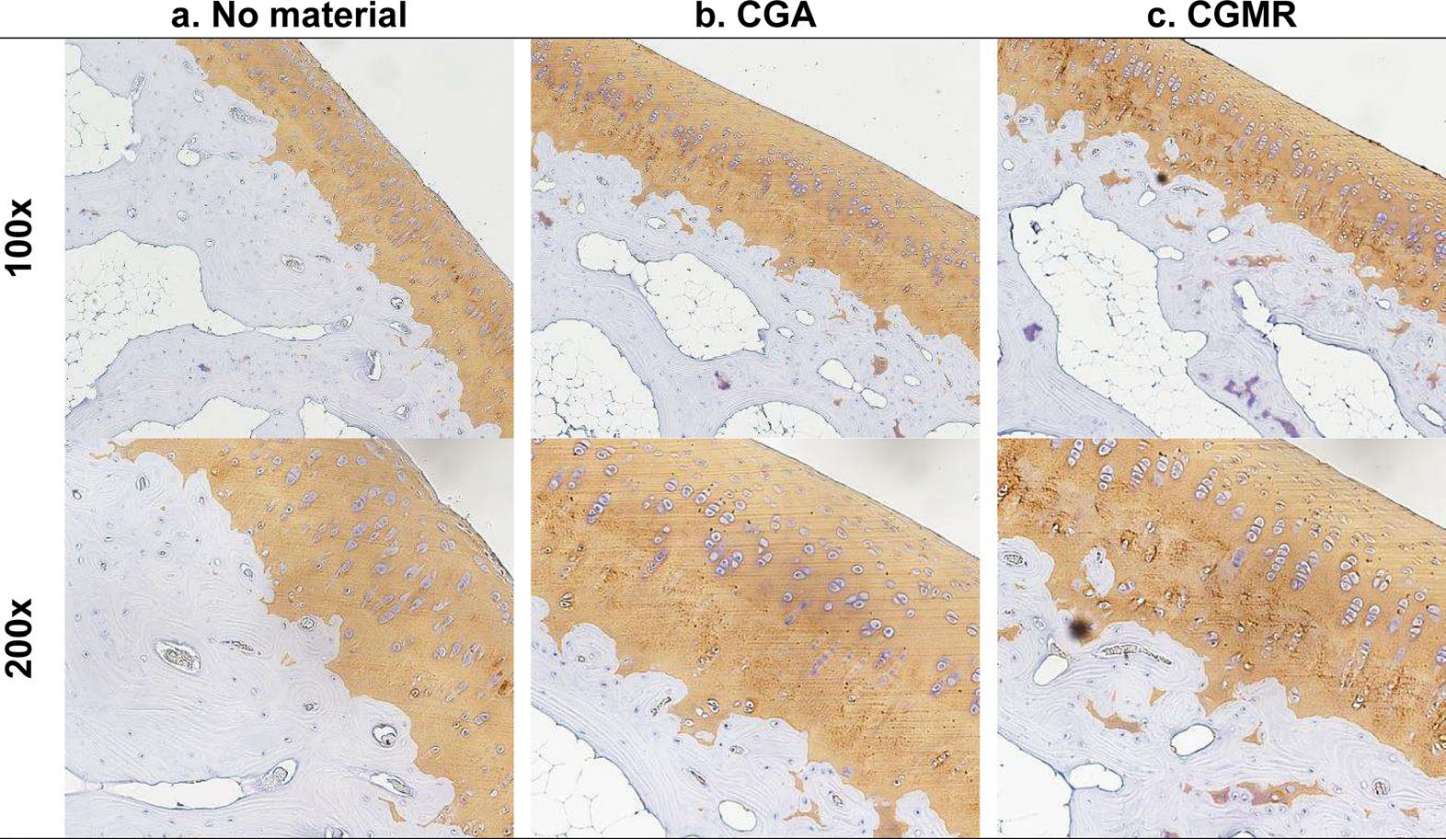
Immunochemical staining for type-II collagen in the regenerated cartilage. Group C exhibits more dense staining than that exhibited by groups A and B

The immunohistochemical staining results for type-II collagen over regenerated cartilage showed heterogeneity in various areas, primarily due to structural irregularity or disruption. We further selected specimens with similar and preferable structural characteristics (modified O’Driscoll score > 20) for evaluation. Figure 4 presents the representative immunochemical staining images for type-II collagen. Staining for type-II collagen was more intense on chondrocytes and the matrix in the adjacent normal cartilage when compared with that in the regenerated area. Groups A, B, and C showed a trend toward an increased intensity of type-II collagen staining in the regenerated cartilage. Moreover, the regenerated cartilage in group C exhibited more dense staining for type-II collagen than groups A and B, especially over the middle and deep cartilage zones. Collectively, these results indicated that cells of regenerated cartilage possess an improved ability to synthesize type-II collagen after chitosan derivative membrane application. However, the selection of a representative image or area from the regenerative cartilage for analysis was limited owing to structural disruption or heterogeneity.

**Fig. 5:**
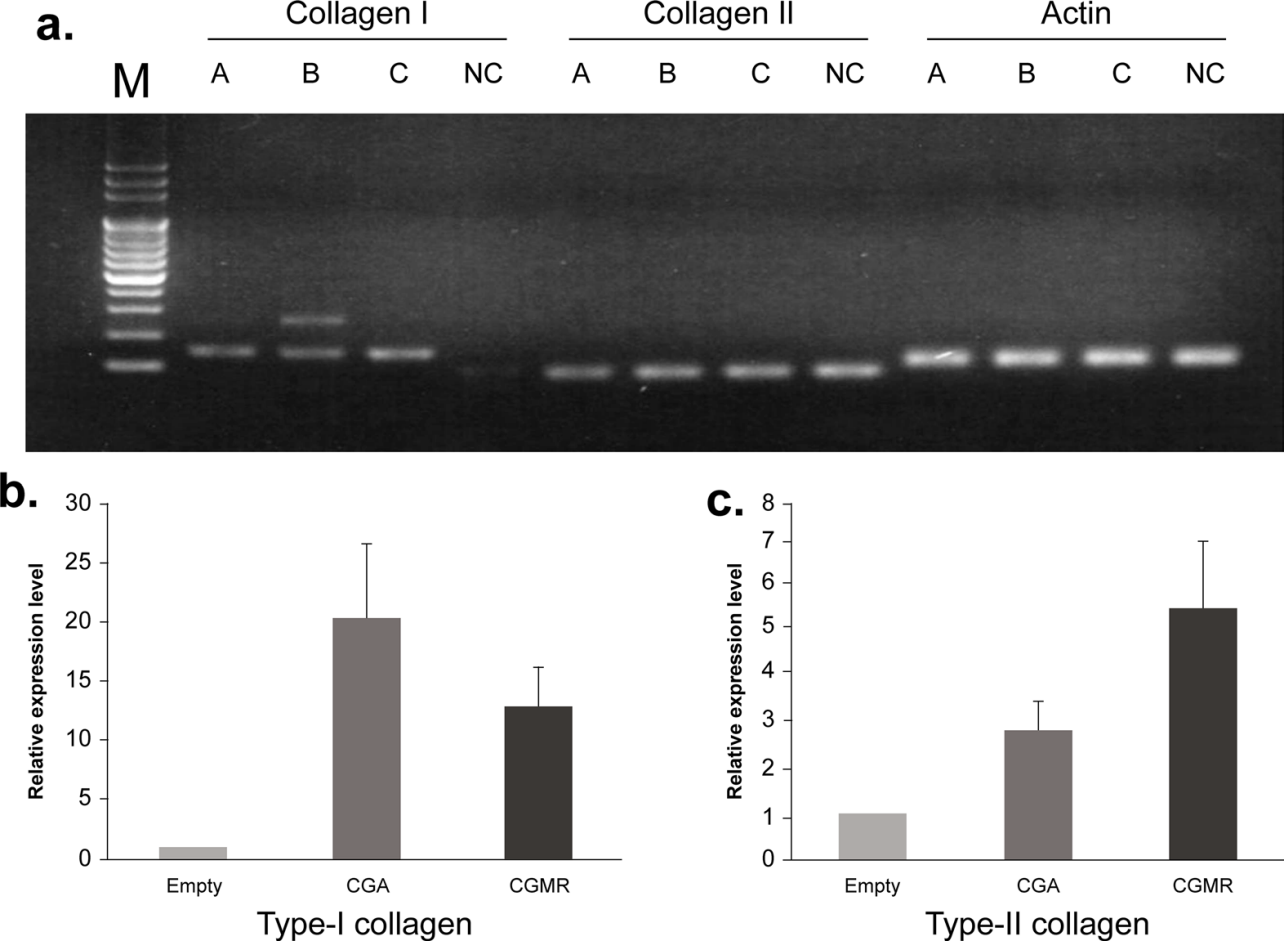
RT-qPCR analysis of mRNA expression for type-I and type-II collagen in the regenerated cartilage. (**a**) RT-qPCR results of type-I and type-II collagen mRNA expression. (**b**) Group B exhibits the highest type-I collagen expression levels, as determined by RT-qPCR. (**c**) Group C exhibits higher mRNA expression of type-II collagen than groups A and B, as determined by RT-qPCR. RT-qPCR, reverse transcription-qualitative polymerase chain reaction

Subsequently, we performed RT-qPCR to confirm type-II collagen mRNA expression in the regenerated cartilage. Specimens for RT-qPCR were pre-surgically assigned at random. In brief, the regenerated cartilage was completely detached or excised for RT-qPCR analysis. Figure 5 presents the RT-qPCR results for type-II collagen. The naive cartilage (NC) from the lateral femoral condyle exhibited the highest expression levels of type-II collagen mRNA, along with traces of type-I collagen expression. Type-I collagen was predominantly expressed in all groups, with group B exhibiting the highest type-I collagen expression levels. Despite group C presenting type-II collagen expression levels twice as high as those of group B (relative mRNA expression level: 5.65 vs. 2.88, respectively), the type-II collagen expression level remained considerably lower than that in the NC (5.65 vs. 15.44). Moreover, RT-qPCR results were consistent with immunohistochemical staining results for type-II collagen expression. Considering the higher type-II collagen expression in group C, our results suggest that the osteochondral defect covered with CGMR may induce superior-quality regenerated cartilage.

## Discussion

Treatment of articular cartilage injury remains one of the most challenging clinical problems in orthopedics. Cartilage defect repair can be stimulated by microfracture surgery, which introduces new cell populations or growth factors into the defect site by penetrating the subchondral bone. The regenerated tissue generally comprises fibrocartilage and has a higher collagen type-I to type-II ratio, making it mechanically inferior to hyaline cartilage [13, 21]. Different strategies have been proposed to improve cartilage regeneration after microfractures. A meta-analysis of animal studies conducted by Pot et al. [14] has revealed that the implantation of biomaterials after bone marrow stimulation could improve the quality of cartilage regeneration. However, there was no difference between natural and synthetic materials in the improvement of cartilage regeneration. Chitosan-based materials were found to elicit a comparable effect to other synthetic biomaterials, including collagen and hyaluronic acid [14]. We have previously shown that glucose adsorption onto chitosan membranes increased human chondrocyte proliferation [19]. This could be attributed to the combined effects of the chitosan membrane and glucose, which is commonly used as an injection material to relieve pain and improve tissue healing in prolotherapy. In the current study, CGA was expected to act as a barrier to attract and maintain fluids and cells, given that it exhibited the effect of glucose for cartilage repair, as observed in prolotherapy when covered over the osteochondral defect. However, the difference in histological scores was not statistically significant in the rabbit model. Conversely, chemical modifications with the glycosylation of chitosan represent an attractive approach, given the improved aqueous solubility improvement and biological relevance exhibited by oligosaccharides. The lactose-modified chitosan obtained from the N-alkylation reaction has a superior ability to induce pig chondrocyte aggregation and stimulate aggrecan and type-II collagen production when compared with the unmodified chitosan [17]. Chitosan-glucose derivatives obtained through the MR have been extensively studied in their fabrication process and characterized by their rheological, chemical, antioxidant, and antimicrobial properties [8–10, 12]. Antioxidants exert positive effects on cartilage protection and regeneration [22, 23]. In addition to the physicochemical properties, the distinct antioxidant property of CGMR may underlie the improved cartilage regeneration observed in the current study. CGMR may create a more appropriate microenvironment for progenitor cells to form a superior histological structure and cell type and induce higher type-II collagen to type-I collagen composition over the regenerated cartilage than CGA or no material.

To the best of our knowledge, this is the first in vivo study to investigate these two glucose-derived chitosan materials. The sample size used here was relatively large, and therefore, the results could be deemed relevant. However, the mechanism underlying the difference in histological scores resulting from the two membranes could not be ascertained, which warrants further investigation owing to the potential of CGMR as an adjuvant therapy for osteochondral defects. Additionally, the trends of type-I and type-II collagen mRNA expression levels were solely presented, and statistical analysis was not performed to determine the significance of the differences among them. This was because only trace mRNA could be isolated from the cartilage specimens using our protocol for individual samples, and we mixed the samples in different groups to obtain the results of quantitative PCR, which was performed a total of five times. Despite the bias in our data, we believe our findings still provide valuable information regarding type-I and type-II collagen composition, and quantitative PCR results corroborated the results of histological and immunohistological assessments.

## Conclusions

Our study validated that CGMR can improve cartilage regeneration in osteochondral defects in a rabbit model. Despite the positive results in vitro, CGA did not exhibit a similar effect in vivo. Therefore, CGMR could be a promising biomaterial and merits further assessment in the field of cartilage regeneration.

## Data Availability

The data that support the findings of this study are available from the corresponding author upon reasonable request.

## Abbreviations

CGA: chitosan membranes with glucose absorption
CGMR: chitosan-glucose derivative membranes obtained via the Maillard reaction
MR: Maillard reaction
EDTA: ethylenediaminetetraacetic acid
COL1A1: collagen type-I alpha 1 chain
COL2A1: collagen type-II alpha 1 chain
RT-qPCR: reverse transcription-qualitative polymerase chain reaction
NC: naive cartilage

## References

[1] Oryan A, Sahvieh S. Effectiveness of chitosan scaffold in skin, bone and cartilage healing. Int J Biol Macromol. 2017;104:1003–11. 28684351 10.1016/j.ijbiomac.2017.06.124

[2] Suh JKF, Matthew HW. Application of Chitosan-based polysaccharide biomaterials in cartilage tissue engineering: a review. Biomaterials. 2000;21:2589–98. 11071608 10.1016/s0142-9612(00)00126-5

[3] Rodríguez-Vázquez M, Vega-Ruiz B, Ramos-Zúñiga R, Saldaña-Koppel DA, Quiñones-Olvera LF. Chitosan and its potential use as a scaffold for tissue engineering in regenerative medicine. Biomed Res Int. 2015. 10.1155/2015/821279. 26504833 10.1155/2015/821279 PMC4609393

[4] LogithKumar R, KeshavNarayan A, Dhivya S, Chawla A, Saravanan S, Selvamurugan N. A review of Chitosan and its derivatives in bone tissue engineering. Carbohydr Polym. 2016;151:172–88. 27474556 10.1016/j.carbpol.2016.05.049

[5] Abarrategi A, Lópiz-Morales Y, Ramos Y, Civantos A, López‐Durán L, Marco F, et al. Chitosan scaffolds for osteochondral tissue regeneration. J Biomed Mater Res A. 2010;95:1132–41. 20878984 10.1002/jbm.a.32912

[6] Medvedeva EV, Grebenik EA, Gornostaeva SN, Telpuhov VI, Lychagin AV, Timashev PS, et al. Repair of damaged articular cartilage: current approaches and future directions. Int J Mol Sci. 2018;19:2366. 30103493 10.3390/ijms19082366 PMC6122081

[7] Sacco P, Cok M, Scognamiglio F, Pizzolitto C, Vecchies F, Marfoglia A, et al. Glycosylated-chitosan derivatives: a systematic review. Molecules. 2020;25:1534. 32230971 10.3390/molecules25071534 PMC7180478

[8] Chung YC, Kuo CL, Chen CC. Preparation and important functional properties of water-soluble chitosan produced through Maillard reaction. Bioresour Technol. 2005;96:1473–82. 15939275 10.1016/j.biortech.2004.12.001

[9] Chung YC, Tsai CF, Li CF. Preparation and characterization of water-soluble chitosan produced by Maillard reaction. Fishs Sci. 2006;72:1096–103.

[10] Affes S, Nasri R, Li S, Thami T, Van Der Lee A, Nasri M, et al. Effect of glucose-induced Maillard reaction on physical, structural and antioxidant properties of chitosan derivatives-based films. Carbohydr Polym. 2021;255:117341. 33436184 10.1016/j.carbpol.2020.117341

[11] Ledl F, Schleicher E. New aspects of the Maillard reaction in foods and in the human body. Angew Chem Int Ed Engl. 1990;29:565–94.

[12] Gullón B, Montenegro MI, Ruiz-Matute AI, Cardelle-Cobas A, Corzo N, Pintado ME. Synthesis, optimization and structural characterization of a chitosan–glucose derivative obtained by the Maillard reaction. Carbohydr Polym. 2016;137:382–9. 26686142 10.1016/j.carbpol.2015.10.075

[13] Buckwalter JA, Mow VC, Ratcliffe A. Restoration of injured or degenerated articular cartilage. J Am Acad Orthop Surg. 1994;2:192–201. 10709009 10.5435/00124635-199407000-00002

[14] Pot MW, Gonzales VK, Buma P, IntHout J, van Kuppevelt TH, de Vries RBM, et al. Improved cartilage regeneration by implantation of acellular biomaterials after bone marrow stimulation: a systematic review and meta-analysis of animal studies. PeerJ. 2016;4:e2243. 27651981 10.7717/peerj.2243 PMC5018675

[15] Mohan N, Mohanan PV, Sabareeswaran A, Nair P. Chitosan-hyaluronic acid hydrogel for cartilage repair. Int J Biol Macromol. 2017;104:1936–45. 28359897 10.1016/j.ijbiomac.2017.03.142

[16] Zhang R, Chang SJ, Jing Y, Wang L, Chen CJ, Liu JT. Application of chitosan with different molecular weights in cartilage tissue engineering. Carbohydr Polym. 2023;314:120890. 37173038 10.1016/j.carbpol.2023.120890

[17] Donati I, Stredanska S, Silvestrini G, Vetere A, Marcon P, Marsich E, et al. The aggregation of pig articular chondrocyte and synthesis of extracellular matrix by a lactose-modified chitosan. Biomaterials. 2005;26:987–98. 15369687 10.1016/j.biomaterials.2004.04.015

[18] Poon L, Wilson LD, Headley JV. Chitosan-glutaraldehyde copolymers and their sorption properties. Carbohydr Polym. 2014;109:92–101. 24815406 10.1016/j.carbpol.2014.02.086

[19] Chang SF, Huang KC, Cheng CC, Su YP, Lee KC, Chen CN, et al. Glucose adsorption to chitosan membranes increases proliferation of human chondrocyte via mammalian target of rapamycin complex 1 and sterol regulatory element-binding protein‐1 signaling. J Cell Physiol. 2017;232:2741–9. 28218386 10.1002/jcp.25869

[20] Frenkel S, Bradica G, Brekke J, Goldman S, Ieska K, Issack P, et al. Regeneration of articular cartilage–evaluation of osteochondral defect repair in the rabbit using multiphasic implants. Osteoarthr Cartil. 2005;13:798–807. 10.1016/j.joca.2005.04.018 15967685

[21] Carballo CB, Nakagawa Y, Sekiya I, Rodeo SA. Basic science of articular cartilage. Clin Sports Med. 2017;36:413–25. 28577703 10.1016/j.csm.2017.02.001

[22] Handl M, Amler E, Bräun K, Holzheu J, Trč T, Imhoff A, et al. Positive effect of oral supplementation with glycosaminoglycans and antioxidants on the regeneration of osteochondral defects in the knee joint. Physiol Res. 2007. 10.33549/physiolres.930917. 16555950 10.33549/physiolres.930917

[23] McAlindon TE, Jacques P, Zhang Y, Hannan MT, Aliabadi P, Weissman B, et al. Do antioxidant micronutrients protect against the development and progression of knee osteoarthritis? Arthritis Rheum. 1996;39:648–56. 8630116 10.1002/art.1780390417

